# Effects of Creatine Supplementation on the Performance, Physiological Response, and Body Composition Among Swimmers: A Systematic Review and Meta-Analysis of Randomized Controlled Trials

**DOI:** 10.1186/s40798-024-00784-8

**Published:** 2024-10-23

**Authors:** Dongxiang Huang, Xiaobing Wang, Tomohiro Gonjo, Hideki Takagi, Bo Huang, Wenrui Huang, Qi Shan, Daniel Hung-Kay Chow

**Affiliations:** 1 School of Physical Education, Shaoguan University, Shaoguan, P.R. China; 2 Department of Health and Physical Education, The Education University of Hong Kong, Hong Kong, P.R. China; 3 Institute for Life and Earth Sciences, School of Energy, Geoscience, Infrastructure and Society, Heriot-Watt University, Edinburgh, UK; 4 Faculty of Health and Sport Sciences, University of Tsukuba, Tsukuba, Japan; 5 School of Physical Education and Sports Science, South China Normal University, Guangzhou, P.R. China; 6 Shenzhen Traditional Chinese Medicine Hospital, Shenzhen, P.R. China

**Keywords:** Creatine, Swimming performance, Body composition

## Abstract

**Background:**

Although recent studies have increasingly focused on examining the potential benefits of creatine supplementation to improve performance in swimming events, the impact of creatine supplementation on swimming performance remains a topic of debate and controversy. A comprehensive meta-analytical review was undertaken to evaluate the effects of creatine supplementation on the performance, physiological response, and body composition among swimmers.

**Methods:**

The research methodology adhered strictly to the guidelines outlined by the Preferred Reporting Items for Systematic Reviews and Meta-Analyses (PRISMA). A comprehensive search was conducted across six databases (Cochrane Library, Web of Science, Scopus, Embase, PubMed, and SPORTDiscus) until March 23, 2024. Eligible studies that investigated the impact of creatine supplementation on swimming time, physiological parameters, and body composition in swimmers were included. For the meta-analysis, a random-effects model was employed to determine the collective effect and assess variations across distinct subgroups defined by swimming time, physiological metrics, and body composition. Meta-regression analysis was conducted on datasets comprising ten or more studies. Standardized mean differences (SMD) along with their corresponding 95% confidence intervals (CI) were calculated. To evaluate the methodological rigor of the included studies, the Physiotherapy Evidence Database (PEDro) scale was utilized.

**Results:**

The systematic review included seventeen studies with a total of 361 subjects. No significant differences were observed in the overall effect during single sprint swimming (SMD: -0.05, 95% CI: -0.26, 0.15; *p* = 0.61), repeated interval swimming (SMD: -0.11; 95% CI: -0.46, 0.25; *p* = 0.56), physiological response (SMD: 0.04, 95% CI: -0.16, 0.23; *p* = 0.71), and body composition (SMD: 0.18; 95% CI: -0.05, 0.41; *p* = 0.12) between creatine and placebo groups.

**Conclusions:**

Creatine supplementation exhibited ineffectiveness in enhancing the performance, physiological response, and body composition among swimmers.

**Supplementary Information:**

The online version contains supplementary material available at 10.1186/s40798-024-00784-8.

## Key Points

- No significant evidence to support that creatine supplementation can improve swimming performance was found.
- Creatine supplementation showed no significant impact on physiological response.
- No significant effects of creatine supplementation on body composition were found.

**Supplementary Information:**

The online version contains supplementary material available at 10.1186/s40798-024-00784-8.

## Background

Creatine (Cr) is pivotal in the modulation of energy metabolism and has been widely used by athletes to enhance muscle mass, strength, and overall sports performance [1, 2]. It has been extensively demonstrated that increasing the dietary intake of Cr leads to elevated levels of Cr and phosphocreatine in skeletal muscles [3]. High-dose short-term (approximately 20 g per day for 5 days) or low-dose long-term (approximately 3 g per day for 28 days) Cr supplementation can result in a roughly 25% increase in muscle Cr and phosphocreatine levels [4, 5]. The dietary incorporation of Cr has the potential to augment muscle Cr and phosphocreatine levels by an estimated range of 20–40% [6].

Swimming is recognized as a sport that requires both aerobic and anaerobic energy systems [7]. The ATP-phosphocreatine energy system plays a crucial role in energy production, contributing 80% during a 50 m swim and 25% during a 100 m swim [8]. The rapid depletion of phosphocreatine in muscles during swimming leads to a substantial increase in energy demand [9, 10]. Consequently, the growing interest in exploring the potential benefits of Cr supplementation for enhancing swimming performance is reflected in the rising number of scientific studies focusing on this area.

Despite numerous studies investigating the impact of Cr supplementation on single sprint swimming [7, 10–17], repeated interval swimming [18–22], or a combination of both [23–25], there is no consensus on its advantages for swimming performance. Most studies suggested that Cr supplementation could not affect single sprint swimming performance [7, 10, 13, 16, 23, 25]. while some studies indicated performance enhancement [12, 14, 15, 17]. Similarly, the impact of Cr on repeated interval swimming performance is a subject of debate while most studies proposed improvement [18, 20, 22–24], Silva et al. (2007) reported no enhancement [21].

Critically evaluating and synthesizing available studies would be useful in understanding the reason behind the inconsistent study outcomes and in generating more concrete evidence on the effect of Cr on swimming performance. Even though Hopwood et al. (2006) conducted a narrative review on this topic [9], their conclusions were based on studies conducted before 2005 and did not address the effects of Cr on physiological indicators and body composition. Since 2005, five studies have explored Cr supplementation’s effects on swimming performance [14–16, 21, 22]. warranting an updated systematic review. Furthermore, given the potential limitations of individual studies, a meta-analysis is crucial for a comprehensive understanding of the conflicting findings.

The objective of this study is to conduct a comprehensive synthesis and meta-analysis of the existing literature regarding the impact of Cr supplementation on the performance, physiological response, and body composition among swimmers.

## Methods

This study evaluated the effects of Cr supplementation on swimming performance, physiological response and body composition, following the protocols outlined in the Preferred Reporting Items for Systematic Reviews and Meta-Analyses (PRISMA) statement [26], as detailed in the electronic supplementary material 1. The study protocol was registered in the Prospective Register of Systematic Reviews (PROSPERO) with the registration number CRD42024536195 prior to conducting the search methodology.

### Literature Search Strategy

The search for relevant studies was conducted independently by two authors (DX and XB), with any discrepancies resolved through third-party adjudication (DC). An exhaustive literature retrieval was conducted across six electronic databases, namely the Cochrane Library, Web of Science, Scopus, Embase, PubMed, and SPORTDiscus, covering the period from their inception to March 23, 2024. Search records were obtained by employing the subsequent Boolean strategy: (Creatine OR “creatine monohydrate” OR “creatine loading” OR “creatine supplementation” OR “oral creatine”) AND (swimming OR swimmers OR swim). The specifics of the search methodology are delineated in Supplementary Table S1, as presented in the electronic supplementary material 2. The search was augmented by manual methods, encompassing an analysis of the reference lists from the selected studies and an assessment of the literature that cited the included research articles (i.e., forward citation tracking).

### Inclusion and Exclusion Criteria

Studies that conformed to the subsequent inclusion criteria were selected for this review: (i) inclusion of swimmers as participants, regardless of their training level, age, or gender; (ii) examination of the isolated effects of Cr ingestion in any form; (iii) provision of detailed information on the protocol of Cr supplementation, including dosage and duration; (iv) the outcomes are swimming time (e.g., 50 m time and 100 m time), physiological indicators (e.g., blood lactate, heart rate, blood ammonia, and blood pH), and body composition (e.g., body mass, lean body mass, and skinfolds); (v) original, peer-reviewed studies written in English; and (vi) utilization of a randomized placebo-controlled design.

Studies were excluded if they: (i) combined Cr with other supplements and could not isolate the effects of Cr; (ii) consisted of comments, editorials, or reviews; (iii) lacked a placebo group for comparison of results; (iv) were conducted on subjects who have a history of a medical ailment, disease, or trauma.; or (v) focused on artistic swimming, water polo, and fin swimming.

### Text Screening

The procedure was carried out independently by two authors (DX and XB), with any differences between the reviewers being reconciled through consensus in consultation with a third author (DC). The initial stage of the process entailed the assessment of titles and abstracts to efficiently narrow down the pool of research studies required to fulfill the inclusion and exclusion criteria. Subsequently, the complete texts were examined by the researchers to identify the experimental trials that were relevant for inclusion.

### Data Extraction

Data extraction from each study that met the inclusion and exclusion criteria encompassed the following: (i) study design details, (ii) characteristics of the study sample, (iii) information on the Cr supplementation protocol (dosage and duration), (iv) specifics of the swimming test protocol, (v) data on swimming performance, physiological response and body composition, and (vi) main findings. Among the included studies, five employed different protocols to assess the same performance outcome [10, 20, 23–25]. In these cases, each protocol was considered an independent dataset for conducting the meta-analysis. Consequently, all protocols were separately included in the relevant analysis of the performance outcome. In instances where essential data were unavailable in the original publication, contact was made with the researchers of the included studies. Information from one article was acquired through direct communication with the authors [13]. Additionally, standard error values provided in certain studies were converted to standard deviation.

### Methodological Quality and Publication Bias

The process was conducted by two separate researchers, DX and XB, with any discrepancies between the reviewers resolved by DC. The methodological rigor of the studies included in the analysis was assessed using the Physiotherapy Evidence Database (PEDro) scale [27]. The PEDro scale is commonly utilized in systematic review studies examining the effectiveness of supplements and nutritional ergogenic resources [28–30]. It provides a reliable and objective means of evaluating the internal validity of randomized controlled trials [31]. The scale consists of 11 items, with ratings assigned to items 2 through 11. A positive response receives a score of 1 point, while a negative response receives 0 points. Thus, the maximum achievable score on the scale is 10 points. A high and low PEDro score indicates a minimal and high risk of bias, respectively. The quality of the PEDro scale was assessed using a scoring system, where scores of 9 or 10 points were considered excellent, scores between 6 and 8 points were considered good, scores between 4 and 5 points were considered fair, and scores of 3 points or lower were considered poor [32]. Potential publication bias across studies was investigated by visually assessing the asymmetry of the funnel plots for the combined data. This method aided in evaluating the presence of bias in the dissemination of research included in the review.

### Statistical Analysis

Standardized mean differences (SMD) and their corresponding 95% confidence intervals (95% CI) were utilized to quantify the effect sizes for the effects of Cr supplements on the performance, physiological response, and body composition in swimmers. The SMD were calculated using pooled mean and standard deviation in the change in the dependent variables from pre- to post-condition for each group, and its values were categorized as trivial (< 0.2), small (0.2–0.3), moderate (0.4–0.8), or large (> 0.8) [33]. Heterogeneity among studies was assessed using the *I*^*2*^ statistic, with low (< 25%), moderate (25–75%), or substantial (> 75%) risk of heterogeneity [34]. To address significant heterogeneity (*I*^*2*^ > 50%), sensitivity analyses were conducted by systematically excluding studies to identify potential outliers or studies with extreme results. This helped assess the robustness of the findings and explore the impact of specific study characteristics, such as swimming distance, on the observed heterogeneity.

Subgroup analyses were carried out to compare the effects of Cr supplementation on different swimming times, specifically 50 m versus 100 m times, to examine variations based on swimming distance. Subgroup analyses were also conducted on various physiological outcomes to differentiate the specific effects of Cr supplementation on different aspects of swimming physiology. In addition, subgroup analyses were also performed on different body composition outcomes, such as body mass and lean body mass, to ascertain if the supplementation has differing effects on various body composition parameters. For all meta-analyses, the random-effects model was applied [35]. Meta-regression analyses assessed potential moderators influencing the impact of Cr supplementation on single sprint swimming during 50 m time trials and blood lactate levels, requiring a minimum of 10 studies for each analysis [36]. Identified moderators comprised performance level (competitive swimmers vs. non-competitive swimmers), duration of supplementation (less than 20 days vs. more than 20 days), and supplementation protocol (acute loading vs. maintenance). Statistical evaluations were conducted using the alpha level set at p < 0.05. Meta analysis was done using Review Manager (5.4.1, USA) software [37], and Meta-regression analyses was conducted using the ‘metan’ command from the Stata (version 15) ‘meta’ package.

## Results

### Search Results

**Fig. 1:**
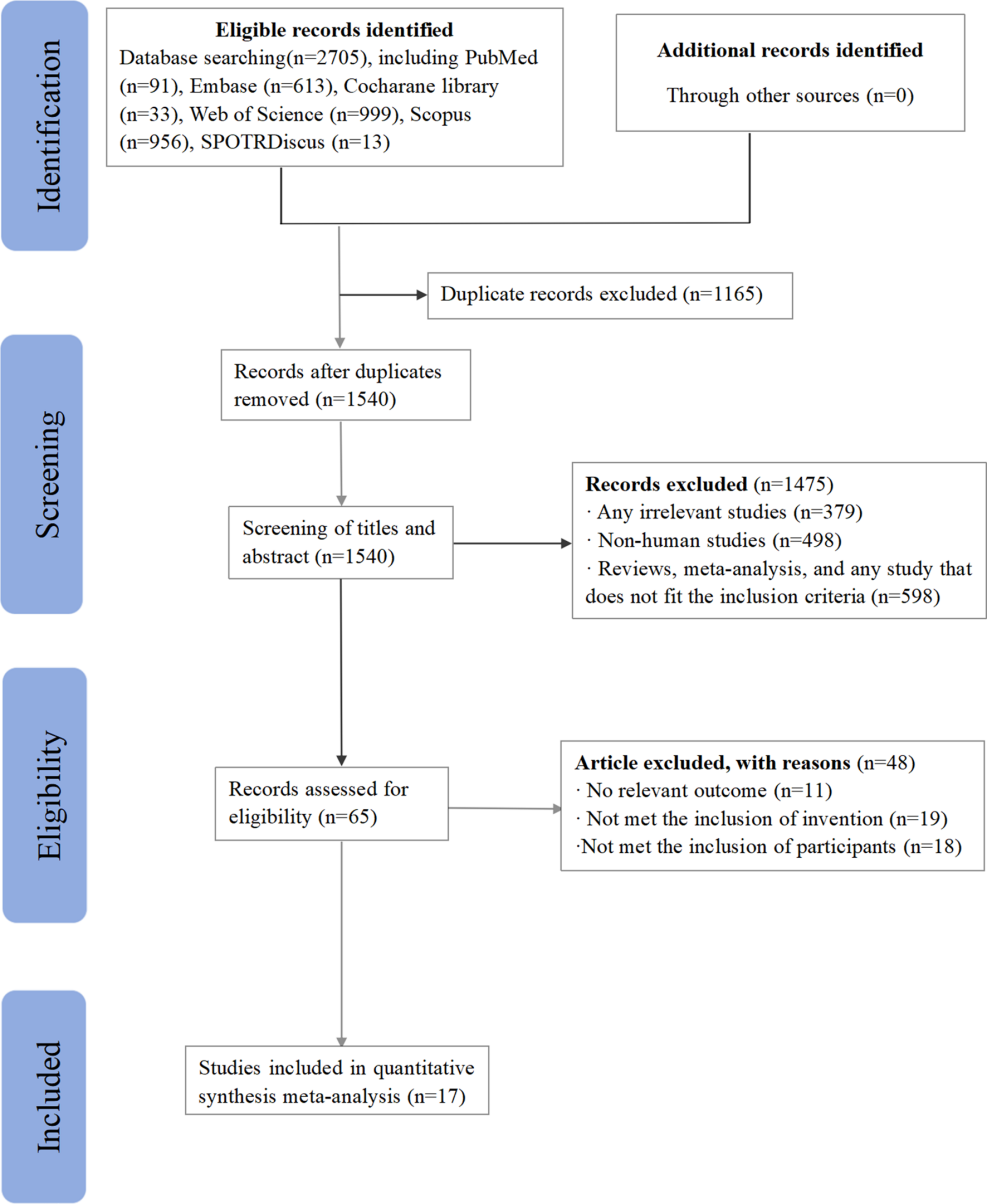
Search strategy flowchart

The systematic search conducted across six databases yielded 2,705 search results. Among these results, 65 full-text papers were carefully examined. After thorough review, 17 studies were deemed eligible and included in the final analysis. Figure 1 presents the flow chart illustrating the search strategy employed.

### Study Characteristics

The meta-analysis included a total of 361 participants across the seventeen studies (Table 1). All studies employed a randomized placebo-controlled design. Among them, twelve studies utilized a double-blind, placebo-controlled, randomized design [11–13, 15, 17–23, 25], while two studies employed a randomized single-blind design [10, 14]. Three studies did not specify the blinding method [7, 16, 24]. The majority of the studies included both male and female participants [10–13, 18–20, 24, 25], although five studies exclusively focused on male swimmers [14, 16, 17, 22, 23] and three targeted female swimmers [7, 15, 21]. The sample size in each study ranged from 10 [7] to 38 [17] participants. Within the studies adhering to the acute loading supplementation regimen, the ingested dosages varied, starting from a low of 0.3 g/kg/bm [13] to a peak of 25 g daily [24]. For the maintenance supplementation regimen, daily dosage consumption fluctuated between 2 g [7] and 20 g [21]. The duration of supplementation protocols ranged from 5 [11, 12, 23] to 63 days [16]. Among the 17 studies, nine investigated the effects of acute loading of Cr supplementation [11, 12, 14, 15, 17, 18, 22, 23, 25], five examined the effects of maintenance loading after an initial acute loading phase [10, 13, 19, 20, 24], and three specifically explored the effects of maintenance loading [7, 16, 21].

The evaluation of swimming performance now clearly delineates swimming time, which consists of single sprint swimming time and repeated interval swimming time, involving distances of 50 m and 100 m. Physiological response includes parameters such as blood lactate levels, heart rate, blood ammonia, and blood pH. Additionally, body composition is evaluated through body mass, lean body mass, and skinfolds.

**Table 1:** Summary of the included studies

		Sample			Supplementation protocol					
Reference	Study design	Sex	Status	N	Acute loading	Maintenance	Duration (days)	Swimming test protocol	Outcomes	Main findings
Azizi. 2011 [15]	RDBPC	F	Competitive swimmers	20	20 g/day	--	6	1 × 60 m	50 m time	Cr alongside an effective conditioning regimen can enhance the athletic performance of female competitive swimmers.
Burke et al. (1996) [11]	RDBPC	M + F	Elite swimmers	32	20 g/day	--	5	1 × 25 m 1 × 50 m 1 × 100 m (swimmers’ preferred strokes)	50 m time 100 m time	Cr supplementation cannot enhance single-effort sprint ability of elite swimmers.
Dawson et al. (2002) [10]	RSBPC	M + F	Competitive junior swimmers	20	20 g/day (5 days)	5 g/day (22 days)	27	1 × 50 m 1 × 100 m (FC swimming)	50 m time 100 m time Blood lactate Body mass Skinfolds	Competitive junior swimmers’ single sprint performance did not improve after 4 weeks of Cr supplementation.
Grindstaff et al. (1997) [18]	RDBPC	M + F	Competitive junior swimmers	18	21 g/day	--	9	3 × 100 m (FC swimming)	50 m time 100 m time Body mass Lean body mass	Competitive junior swimmers may benefit from 9 days of Cr supplementation during sprint swimming.
Leenders et al. (1999) [19]	RDBPC	M + F	Competitive university swimmers	32	20 g/day (6 days)	10 g/day (8 days)	14	6 × 50 m 10 × 25 m (swimmers’ preferred strokes)	Body mass Lean body mass	The impact of repeated intervals during training on performance in a single competitive event remains uncertain.
Mendes et al. (2004) [25]	RDBPC	M + F	Competitive swimmers	18	20 g/day	--	8	1 × 100 m 1 × 50 m 3 × 3 × 50 m (swimmers’ preferred strokes)	50 m time 100 m time Blood lactate	Supplementing with Cr does not boost performance or muscle mass in swimmers.
Mujika et al. (1996) [12]	RDBPC	M + F	Competitive swimmers	20	20 g/day	--	5	1 × 25 m 1 × 50 m 1 × 100 m	50 m time 100 m time Blood lactate Body mass Blood ammonia	Highly trained swimmers cannot benefit from Cr supplementation for sprint performance.
Peyrebrune et al. (1998) [23]	RDBPC	M	Competitive swimmers	14	9 g/day	--	5	1 × 50 yards 8 × 50 yards	50 yards time Heart rate Blood lactate Blood ammonia Blood pH	Daily intake of 9 g Cr for 5 days improves swimming performance in elite athletes during repeated sprints, but not in a single 50-yard sprint.
Peyrebrune et al. (2005) [20]	RDBPC	M + F	Competitive swimmers	20	20 g/day (10 days)	3 g/day (42 days)	52	2 × (5 x 200 m) 3 × (8 x 50 m)	50 m time heart rate Blood lactate Blood ammonia Body mass Skinfolds	Oral Cr supplementation increases repeated sprint swimming performance.
Roshan et al. (2013) [22]	RDBPC	M	Non-elite swimmers	16	20 g/day	--	6	6 × 50 m (FC swimming)	Blood lactate Heart rate	Swimming performance, blood lactate levels, anaerobic performance, and heart rate fluctuations may improve with Cr supplementation.
Scorcine et al. 2013 [16]	RPC	M	trained swimmers	30	--	5 g /day	63	1 × 500 m 1 × 50 m	50 m time	Cr supplementation had no impact on endurance in swimming
Selsby et al. (2003) [13]	RDBPC	M + F	Competitive university swimmers	15	0.3 g/ kg /bm (5 days)	2.25 g/day (9 days)	14	1 × 50 yards 1 × 100 yards	50 yards time 100 yards time Body mass	Cr supplementation for swimming works for 50- and 100-yard sprints.
Sompol Sanguanrungsirikul. 2004 [17]	RDBPC	M	trained swimmers	38	10 g/day	--	7	1 × 400 m	50 m time	Cr supplementation in young amateur swimmers enhanced anaerobic capacity, power, and performance in the final 50-meter sprint of the 400-meter competitive event.
Silva et al. (2007) [21]	RDBPC	F	Competitive swimmers	16	--	20 g/day	21	2 × 25 m	Body mass Lean body mass	Cr supplementation did not affect weight, composition, or performance.
Thompson et al. 1996 [7]	RPC	F	university swimmers	10	--	2 g/day	42	1 × 100 m 1 × 400 m	50 m time	Oral Cr supplementation does not affect muscle Cr concentration, oxygen supply, or aerobic/anaerobic metabolism.
Theodorou et al. (1999) [24]	RPC	M + F	Elite swimmers	22	25 g /day (4 days)	5 g /day (56 days)	60	A = 10 × 50 m B = 8 × 100 m C = 15 × 100 m (swimmers’ preferred distances)	50 m time 100 m time Body mass	Swimming performance improves dramatically after 4 days of acute Cr loading.
Vatani et al. (2011) [14]	RSBPC	M	Amateur swimmers	20	20 g /day	--	6	1 × 50 m 1 × 100 m (breaststroke)	50 m time 100 m time	Short-term Cr supplementation increased amateur swimmer 50 m sprint performance.

F: female. FC: front crawl. M: male. RDBPC: randomized double-blind placebo control. RSBPC: randomized single-blind placebo control. RPC: randomized placebo control

### Methodological Quality and Publication Bias

The average PEDro scale score for the studies included was 8.41, indicating a high level of methodological quality. Out of the seventeen studies, twelve [11–13, 15, 17–23, 25] were classified as excellent quality, while five investigations [7, 10, 14, 16, 24] were categorized as good quality. The detailed scores on the PEDro scale can be found in Table 2. Figure S1 in Supplementary Material 2 displays the publication biases across various outcomes, encompassing single sprint swimming time, repeated interval swimming time, physiological variables, and body composition metrics. The scatter of data points forms an approximate inverted funnel pattern around the overall effect size, suggesting no publication bias.

**Table 2:** Ratings of the included studies based on the Physiotherapy evidence database (PEDro) scale

Study	1	2	3	4	5	6	7	8	9	10	11	Total
Azizi. 2011 [15]	Yes	1	0	1	1	1	1	1	1	1	1	9
Burke et al. (1996) [11]	Yes	0	1	1	1	1	1	1	1	1	1	9
Dawson et al. (2002) [10]	Yes	1	1	1	1	0	0	1	1	1	1	8
Grindstaff et al. (1997) [18]	Yes	0	1	1	1	1	1	1	1	1	1	9
Leenders et al. (1999) [19]	Yes	1	1	1	1	1	1	0	1	1	1	9
Mendes et al. (2004) [25]	Yes	1	0	1	1	1	1	1	1	1	1	9
Mujika et al. (1996) [12]	Yes	1	0	1	1	1	1	1	1	1	1	9
Peyrebrune et al. (1998) [23]	Yes	1	0	1	1	1	1	1	1	1	1	9
Peyrebrune et al. (2005) [20]	Yes	1	0	1	1	1	1	1	1	1	1	9
Roshan et al. (2013) [22]	Yes	1	0	1	1	1	1	1	1	1	1	9
Scorcine et al. 2013 [16]	Yes	1	0	0	0	0	1	1	1	1	1	6
Selsby et al. (2003) [13]	Yes	1	1	1	1	1	1	1	1	1	1	10
Sompol Sanguanrungsirikul. 2004 [17]	Yes	1	0	1	1	1	1	1	1	1	1	9
Silva et al. (2007) [21]	Yes	1	1	1	1	1	1	1	1	1	1	10
Thompson et al. 1996 [7]	Yes	1	0	0	0	0	1	1	1	1	1	6
Theodorou et al. (1999) [24]	Yes	1	0	1	0	0	0	1	1	1	1	6
Vatani et al. (2011) [14]	Yes	1	0	1	1	0	0	1	1	1	1	7

1, clear eligibility criteria were established; 2, volunteers were randomly assigned to their respective groups; 3, allocation was concealed; 4, baseline characteristics of the groups were similar in terms of important prognostic indicators; 5, blinding was implemented for all participants; 6, therapists administering the therapy were also blinded; 7, assessors measuring key outcomes were blinded; 8, outcome measures were obtained from 85% of the initially allocated participants; 9, all participants with available outcome measures received the allocated treatment or control condition, or an intention-to-treat analysis was conducted; 10, Statistical analyses comparing groups were presented for a minimum of one primary outcome; 11, The research furnished point estimates as well as variability indices for at least one primary outcome

### Meta-analysis Results

#### Single Sprint Swimming Time

**Fig. 2:**
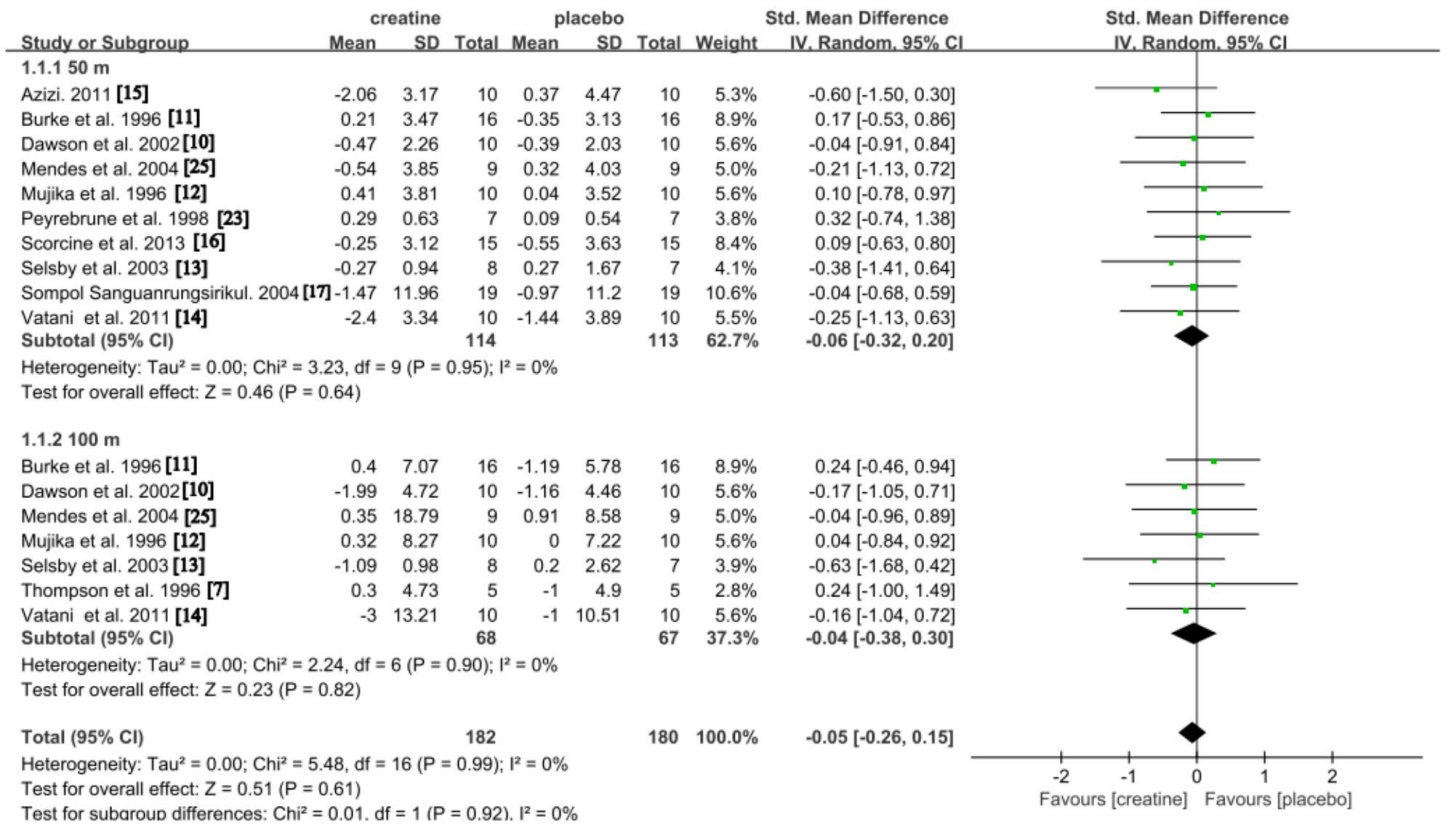
Pooled analysis of the effect of Cr on single sprint swimming time. Subgroup effects are delineated according to the categorization variables associated with time across varying distances (50 m time vs. 100 m time)

A total of eleven studies reported the effect of Cr on single sprint swimming time [7, 10–17, 23, 25]. As depicted in Fig. 2, the results of our meta-analysis indicate that there was no overall effect favoring the Cr condition (SMD: -0.05, 95% CI: -0.26, 0.15; *p* = 0.61), with low but non-significant heterogeneity observed *(p* = 0.99; *I*^*2*^ = 0%). Subgroup analysis further revealed that there was a trivial and non-significant reduction observed in the 50 m time (SMD: -0.06; 95% CI: -0.32, 0.20; *p* = 0.64), with low but not significant heterogeneity detected (*p* = 0.95; *I*^*2*^ = 0%). Additionally, a trivial but insignificant improvement was found in the 100 m time (SMD: -0.04; 95% CI: -0.38, 0.30; *p* = 0.82), with no significant heterogeneity observed (*p* = 0.90; *I*^*2*^ = 0%).

#### Repeated Interval Swimming Time

**Fig. 3:**
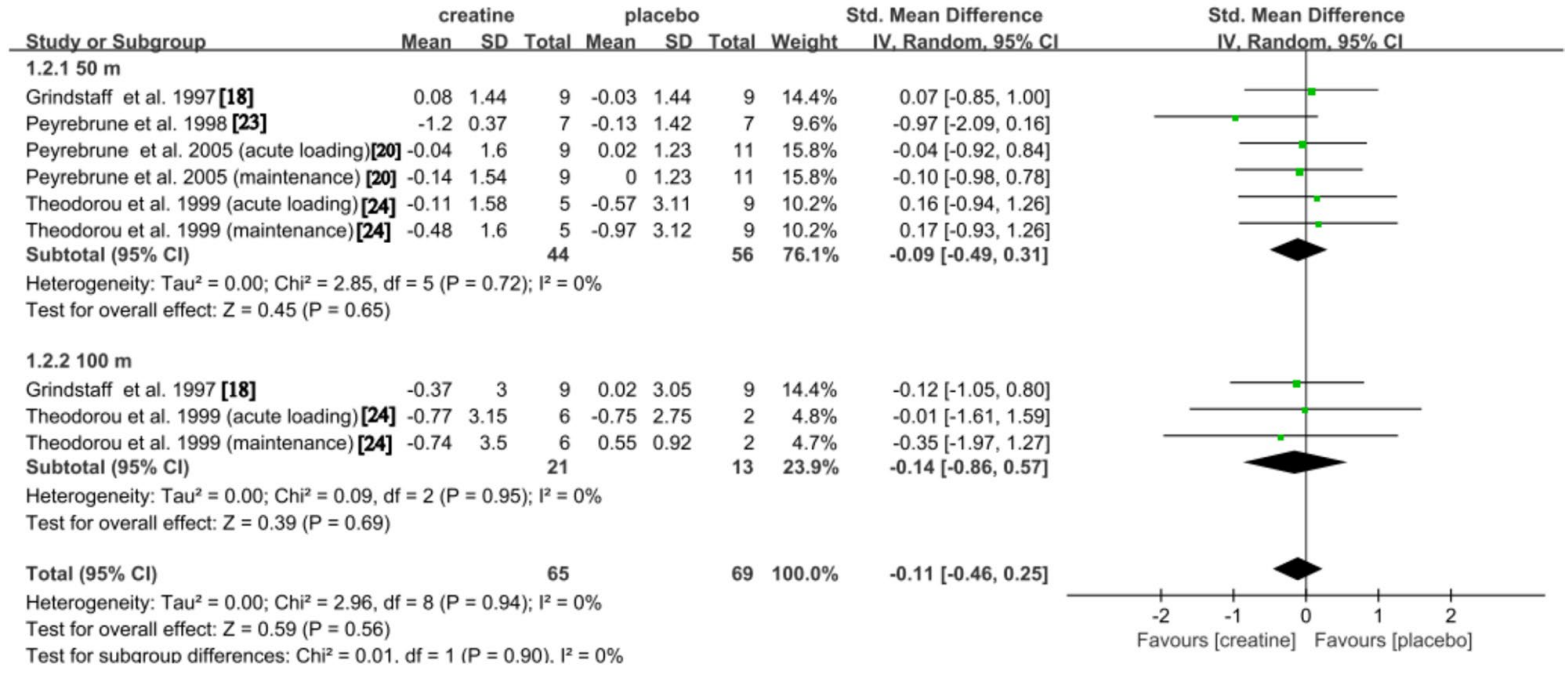
Pooled analysis of the effect of Cr on repeated interval swimming time. Subgroup effects are delineated according to the categorization variables associated with time across varying distances (50 m time vs. 100 m time)

The impact of Cr on repeated interval swimming time was examined in a cumulative four studies [18, 20, 23, 24]. Compared to placebo, supplementation with Cr led to a trivial and not significant reduction in the overall effect (SMD: -0.11; 95% CI: -0.46, 0.25; *p* = 0.56), with low but not significant heterogeneity observed (*p* = 0.94; *I*^2^ = 0%). Subgroup analysis further indicated a trivial and not significant decrease in repeated interval swimming time during the 50 m test (SMD: -0.09; 95% CI: -0.49, 0.31; *p* = 0.65), with low and not significant heterogeneity detected (*p* = 0.72; *I*^*2*^ = 0%). Similarly, a trivial and non-significant effect was found for the 100 m test (SMD: -0.14; 95% CI: -0.86, 0.57; *p* = 0.69), with no significant heterogeneity observed (*p* = 0.95; *I*^*2*^ = 0%) (Fig. 3).

#### Physiological Response

**Fig. 4:**
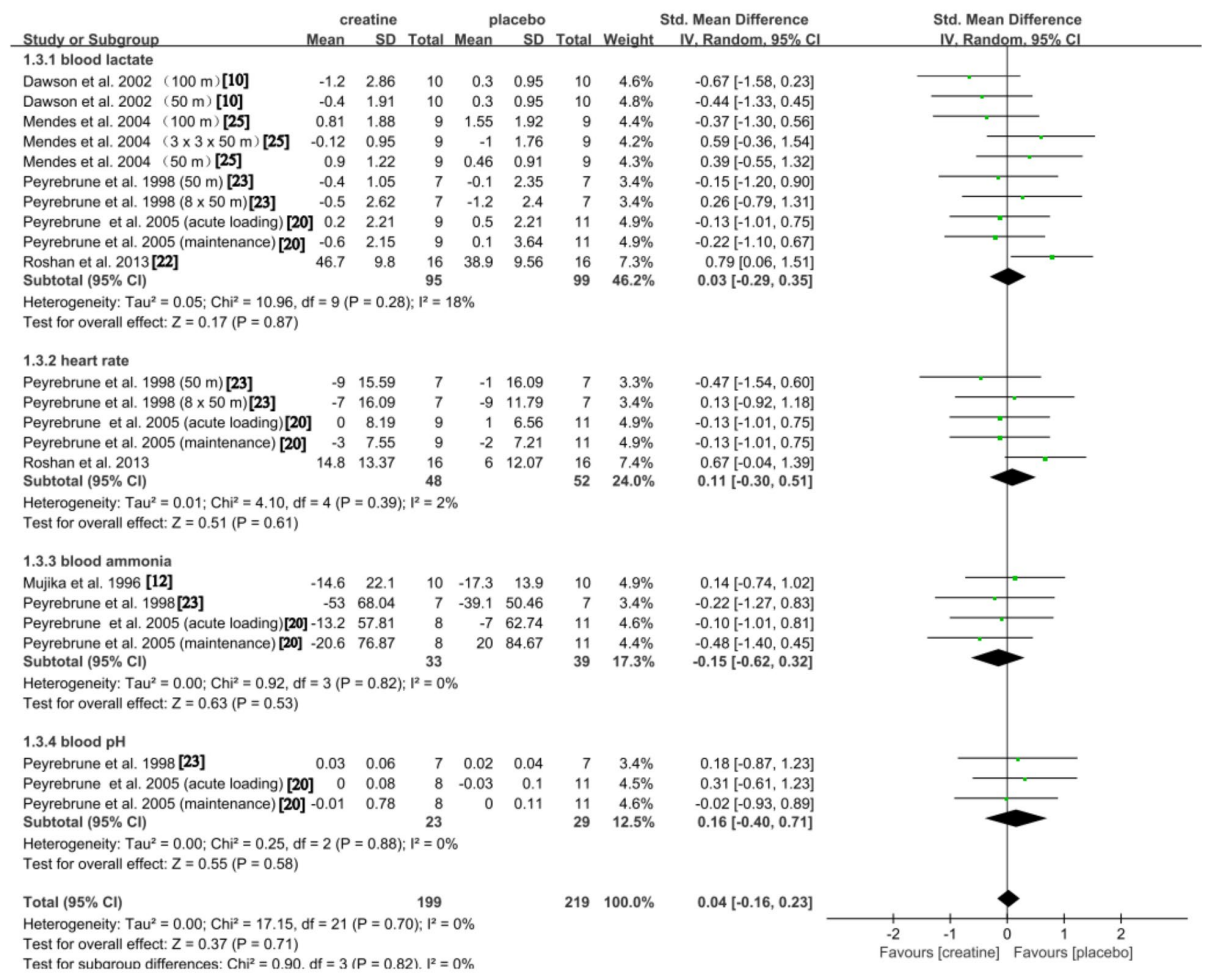
Pooled analysis of the effect of Cr on physiological variables. Subgroup effects are delineated according to the categorization variables associated with different outcomes (blood lactate vs. heart rate vs. blood ammonia vs. blood pH)

Six studies examined the impact of Cr on physiological factors [10, 12, 20, 22, 23, 25]. In the meta-analysis, no significant difference was found for the total effect when comparing the Cr group to the placebo group (SMD: 0.04, 95% CI: -0.16, 0.23; *p* = 0.71), with no significant heterogeneity observed (*p* = 0.70; *I*^*2*^ = 0%). Cr supplementation showed a trivial and non-significant effect on blood lactate levels (SMD: 0.03; 95% CI: -0.29, 0.35; *p* = 0.87), with no significant heterogeneity detected (*p* = 0.28; *I*^*2*^ = 18%). Furthermore, no significant effect was observed on heart rate (SMD: 0; 11; 95% CI: -0.30, 0.51; *p* = 0.61), and no significant heterogeneity was found (*p* = 0.39; *I*^*2*^ = 2%). Additionally, no significant negative effect was found on blood ammonia levels (SMD: -0.15, 95% CI: -0.62, 0.32; *p* = 0.53), with no significant heterogeneity observed (*p* = 0.82; *I*^*2*^ = 0%). Moreover, trivial and non-significant improvement were observed in blood pH (SMD: 0.16, 95% CI: -0.40, 0.71; *p* = 0.58), with no significant heterogeneity detected (*p* = 0.88; *I*^*2*^ = 0%) (Fig. 4).

#### Body Composition

**Fig. 5:**
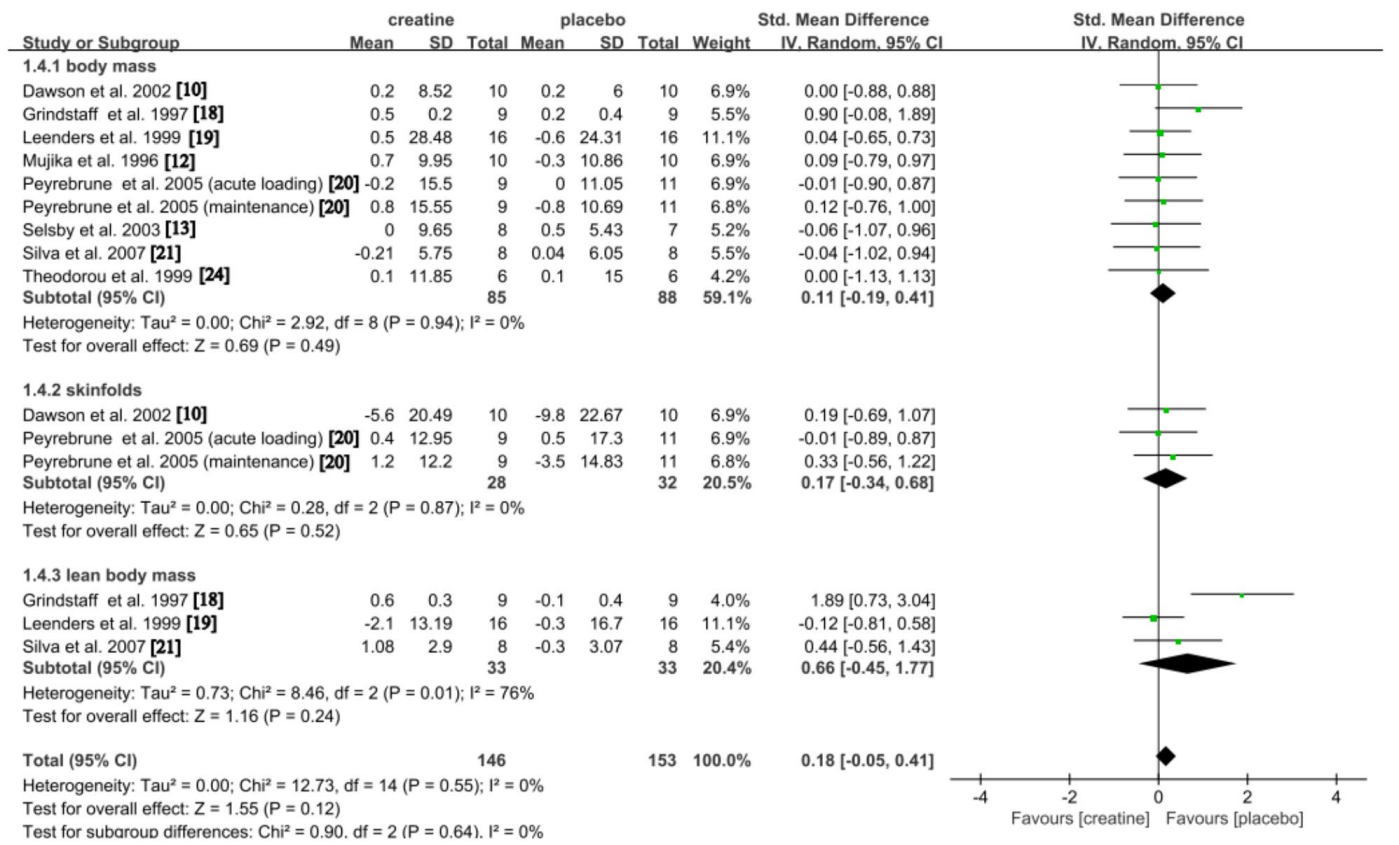
Pooled analysis of the effect of Cr on body composition. Subgroup effects are delineated according to the categorization variables associated with different outcomes (body mass vs. skinfolds vs. lean body mass)

The influence of Cr on body composition was investigated in seven studies [10, 12, 13, 18–20, 24]. In comparison to the placebo, there was a trivial and not statistically significant difference observed for the overall effect (SMD: 0.18; 95% CI: -0.05, 0.41; *p* = 0.12), with a low and non-significant level of heterogeneity detected (*p* = 0.55; I2 = 0%). A trivial and not statistically significant increase was observed in body mass (SMD: 0.11; 95% CI: -0.19, 0.41; *p* = 0.49), with a low and non-significant level of heterogeneity detected (*p* = 0.94; I2 = 0%). Similarly, Cr demonstrated trivial and not significant effect in body composition as measured by skinfolds (SMD: 0.17; 95% CI: -0.34, 0.68; *p* = 0.52), with a low and non-significant level of heterogeneity detected (*p* = 0.87; I2 = 0%). On the other hand, moderate but not statistically significant increases were observed in lean body mass (SMD: 0.66; 95% CI: -0.45, 1.77; *p* = 0.24), with a substantial but non-significant level of heterogeneity detected (*p* = 0.01; *I*^*2*^ = 76%) (Fig. 5). The study conducted by Grindstaff et al. (1997) resulted in high risk of heterogeneity on lean body mass [18]. Subsequent to the exclusion of this particular study, a meta-analysis was performed on the remaining data, as shown in Fig. S2 (supplementary material 2), which led to a considerable decrease in heterogeneity (from 76 to 0%). However, the variation between groups did not reveal a statistically significant disparity when comparing the Cr group with the placebo group.

### Meta-Regression Results

Table 3 presents the results of a meta-regression analysis assessing the impact of Cr on single sprint swimming time during 50 m event (single 50 m time) and blood lactate levels. The analysis considered moderators such as performance level (competitive swimmers vs. non-competitive swimmers), duration of supplementation (less than 20 days vs. more than 20 days), and supplementation protocol (acute loading vs. maintenance). Findings indicated that these moderators did not significantly affect the single 50 m time and blood lactate.

**Table 3:** Meta-regression analysis for different moderators to predict the effects of cr supplementation on single 50 m time and blood lactate

	Coefficient	Standard error	Z	*P* value	95% CI
**Single 50 m time**					
Performance level	0.06	0.31	0.19	0.85	-0.55, 0.67
Duration of supplementation	0.41	0.62	0.66	0.51	-0.81, 1.63
Supplementation protocol	-0.33	0.55	-0.59	0.55	-1.41, 0.75
**Blood lactate**					
Performance level	0.71	0.42	1.69	0.09	-0.11, 1.53
Duration of supplementation	-0.56	0.33	-1.69	0.09	-1.21, 0.09
Supplementation protocol	-0.56	0.33	-1.69	0.09	-1.21, 0.09

## Discussion

### Summary of Main Findings

This study presents the first systematic review and meta-analysis investigating the effects of Cr supplementation on the performance (measured as 50 m time, 100 m time), physiological response (measured as heart rate, blood lactate, blood ammonia, and blood pH) and body composition (measured as body mass, lean body mass, and skinfolds) in swimmers. The main finding was that there was no statistically significant impact of Cr supplementation on the performance, physiological response and body composition among swimmers. Collectively, the findings of this meta-analysis indicate that incorporating Cr supplementation might not be a viable consideration for swimmers seeking to enhance their swimming performance. These results inform and direct future research efforts to further explore the ergogenic effects of Cr in this specific athletic context.

### Single Sprint Swimming

Our meta-analysis demonstrated that Cr supplementation did not significantly impact 50 m and 100 m times in single sprint swimming (Fig. 2). This finding aligns with previous studies [10–12, 23, 25], likely due to the relatively lower depletion of phosphocreatine stores during single-sprint swimming. Therefore, Cr supplementation shows limited effectiveness in this context.

However, contrasting results were reported by two studies [13, 14], where significant improvements in 50 m sprint times were observed following Cr supplementation. These discrepancies were attributed to variations in participant athletic abilities, study designs, and exercise protocols [13]. To address these inconsistencies, we conducted a meta-regression analysis examining factors such as the swimmers’ performance level, the duration of Cr supplementation, and the supplementation protocol. Our analysis revealed that none of these factors significantly influenced the effectiveness of Cr supplementation on 50 m sprint swimming performance (Table 3).

Similarly, no significant effect on 100 m swimming performance was observed (Fig. 2), consistent with previous studies [10–12, 14, 25]. Interestingly, improvements in performance in the single 100 m freestyle sprint were demonstrated by Selsby et al. (2003) [13]. The authors attributed these improvements to the performance level of the participants, who had lower swimming abilities and levels. They suggested that lower-level swimmers may be more likely to increase intramuscular Cr stores than elite athletes. However, this viewpoint was not supported by Vatani et al. (2011) [14], which also included participants with lower-level swimming abilities. Sensitivity analysis identified Selsby et al. (2003) [13] as a source of heterogeneity, likely due to participant ability and sample size. Excluding this study reduced heterogeneity significantly, confirming that Cr supplementation is unlikely to enhance single-sprint swimming performance.

In conclusion, the comprehensive findings from our meta-analysis, including the additional insights gained from our meta-regression and sensitivity analyses, indicate that Cr supplementation does not consistently or significantly enhance single-sprint swimming performance across various levels of swimmers. While isolated reports suggest some improvements in specific subgroups, these were not sufficiently consistent to confirm a general benefit across a diverse range of swimmers. Our study underscores the need to consider individual variability and study design when assessing the efficacy of nutritional supplements in enhancing sports performance. Future research should continue to explore the nuanced effects of Cr under more defined and controlled conditions to ascertain any specific advantages it might offer to particular populations.

### Repeated Interval Swimming

While anaerobic energy significantly contributes to a single 50 m sprint, accounting for up to 80% of energy expenditure [38], its role diminishes as repetitions increase during repeated interval swimming, with aerobic metabolism becoming more prominent [7], especially as phosphocreatine stores are depleted, even with supplementation. Although phosphocreatine resynthesis remains crucial for short bursts of effort, maintaining performance across multiple sprints increasingly relies on aerobic pathways [39]. The potential benefits of Cr supplementation for anaerobic exercise performance have been established [40], particularly in enhancing phosphocreatine resynthesis after sprints [41–43]. However, in repeated sprint swimming, its impact may be limited by the growing reliance on aerobic metabolism as phosphocreatine stores are depleted. Numerous studies have suggested that Cr can enhance performance in repeated interval swimming during both 50 m time [18, 20, 23, 44] and 100 m time [23]. However, the physiological mechanisms underlying these improvements remain unclear, with many researchers still uncertain about the reasons behind this effect.

Our meta-analysis revealed a novel finding that Cr supplementation did not significantly affect 50 m time, 100 m time, or overall time in repeated interval swimming (Fig. 3). This result aligns with Fernández-Landa et al. (2023) meta-analyses, which indicated that Cr supplementation had no significant impact on endurance performance [45]. This might be due to Cr’s action at the peripheral muscle level. While Cr is known to enhance muscle hypertrophy and increase the recruitment of fast-twitch muscle fibers [46], these alterations in skeletal muscle could potentially negatively affect endurance performance. Furthermore, Leenders et al. (1999) investigated the effects of Cr supplementation on average velocity during a 10 × 25 yard repeated interval swim set and found no significant change. The authors suggested that this lack of effect is likely due to the short recovery period between sprints, which may have been insufficient for adequate replenishment of phosphocreatine stores [19].

Although our analysis revealed no statistically significant enhancements in 50 m and 100 m repeated interval swimming times following Cr supplementation, these results should be interpreted with caution due to the small sample sizes and varying quality of the included studies. In particular, our conclusions may be influenced by biases related to not accounting for differences in training setups and rest intervals. Future research should rigorously control experimental variables, such as the number of repetitions and the duration of rest intervals, to ensure more reliable results. It is also critical to increase the sample size and improve the overall quality of studies to address these limitations effectively.

### Physiological Response

A distinctive aspect of this review is the use of meta-analysis to investigate the impact of Cr on physiological markers in swimmers. Our findings indicate that Cr supplementation does not significantly alter post-exercise blood lactate levels, heart rate, blood ammonia, or pH values, as illustrated in Fig. 4. These outcomes are consistent with those reported by Peyrebrune et al. (1998, 2005) [20, 23].

A study reported a similar lactate concentrations before and after supplementation [25]. However, other studies presented contrasting views, suggesting that Cr supplementation could improve lactate levels [10, 22]. While the underlying mechanisms were not clearly elucidated in these studies, a research suggested that Cr may delay lactate accumulation associated with sprint swimming [13]. Additional findings from Roshan et al. demonstrated an improvement in heart rate with Cr supplementation [22].

Regarding blood ammonia levels, two studies proposed views that contradict our findings, suggesting that Cr supplementation significantly reduced post-exercise blood ammonia levels [12, 47]. They attributed this phenomenon to an enhanced rate of ADP to ATP formation after Cr supplementation, leading to a decreased degradation of purine nucleotides. Although research on the effects of Cr supplementation on physiological indicators yields inconsistent results, with some studies indicating no significant changes and others suggesting improvements, our meta-analysis concludes that Cr supplementation has no impact on these indicators. Nevertheless, given the limited scope of studies incorporated, our results ought to be approached with prudence. Future research should further investigate Cr’s potential effects on swimmers’ physiological indicators, emphasizing the inclusion of more diverse sample sizes and supplementation regimes to enhance the robustness of the evidence base.

### Body Composition

Although Cr supplementation has been shown to increase body mass in various sports [48–58], our meta-analysis of the effects of Cr on body composition in swimmers found no significant changes in body mass and lean body mass with Cr supplementation (Fig. 5). These findings are consistent with previous research [18, 19]. some studies also reported no significant effect of Cr on body mass [10, 13, 24]. Mujika et al. (1996) [12], however, reported a significant increase in body mass following Cr supplementation. Mujika et al. (1996) and Grindstaff et al. (1997) measured body mass immediately after acute loading [12, 18], while other studies measured body mass after an acute loading followed by a maintenance phase [10, 13, 19, 20, 24]. It is worth noting that Grindstaff et al. (1997) [18] had a more extended Cr supplementation period of 9 days compared to the 5-day supplementation period in Mujika et al. (1996) [12]. These differences in the measurement timing or supplementation duration may explain the inconsistent conclusions between Mujika et al. (1996) [12] and other studies [10, 13, 18–20, 24].

Subgroup analysis exhibited no significant effects of Cr on skinfolds (Fig. 5). This is consistent with Dawson et al. (2002) [10] and Grindstaff et al. (1997) [18], who found no impact of Cr on skinfolds. However, it is pertinent to acknowledge that this analysis was based on only three studies. Further research is necessary to determine the impact of Cr supplementation on body composition in swimmers.

### Limitations and Methodological Quality

The primary limitation of this systematic review and meta-analysis is the limited number of studies, totaling 17, examining creatine supplementation in swimmers. This small sample necessitated the inclusion of studies with combined male and female data, potentially obscuring sex-specific effects. Our meta-regression analysis was also confined to a few covariates: performance level, duration of supplementation, and protocol, which only affected the 50 m time in single sprint swimming and blood lactate levels. Additionally, these results may be biased due to variations in training setups and rest intervals across studies. To enhance the reliability of future research, it is crucial to increase the sample size and improve the methodological quality of studies. Moreover, future studies should separate male and female data to better understand sex-specific responses and apply stringent controls on experimental variables, including repetition counts and rest periods, to mitigate these limitations effectively.

From a methodological perspective, the included studies exhibited good to excellent quality according to the PEDro scale. This indicates that included studies with suboptimal research methodology did not compromise the current meta-analysis results. As a result, the validity of our conclusions is strengthened, as studies of inadequate methodological quality do not influence the findings presented in this study.

## Conclusions

The impact of Cr on swimming is minimal and lacks statistical significance, suggesting that it is unlikely to be an effective enhancer of swimming performance. Considering the prevalence of this supplement as one of the most commonly utilized ergogenic aids in swimming, it is crucial for athletes, coaches, nutritionists, dietitians, and sports scientists to carefully consider the findings of the current meta-analysis, especially when seeking to optimize swimming performance.

Future research should focus on exploring the benefits of Cr supplementation in specific populations, such as international, national, regional, recreational, and lower-level swimmers, and understanding its potential side effects. Investigating which subgroups are more likely to benefit from Cr supplementation and examining side effects such as tremors, insomnia, increased heart rate, headaches, abdominal/gastrointestinal discomfort, and muscle soreness will be crucial. Conducting these studies will contribute to a more comprehensive understanding of the potential role of Cr in swimming performance.

## Electronic Supplementary Material

Below is the link to the electronic supplementary material.

Supplementary Material 1

Supplementary Material 2  (Please replace 'Supplementary Material 2’ by the newly uploaded file 'Supplementary Material_2.pdf’ as there are changes in the reference numbers)

## Data Availability

The datasets used/analysed during the current study are available from the corresponding author on reasonable request.

## Abbreviations

Cr: Creatine
CI: Confidence interval
PRISMA: The preferred reporting items for systematic reviews and meta-analysis
SD: Standard deviation
F: Female
FC: Front crawl
M: Male
RDBPC: Randomized double-blind placebo control
RSBPC: Randomized singles-blind placebo control
RPC: Randomized placebo control
