# Supplementary material for: Effects of Creatine Supplementation on the Performance, Physiological Response, and Body Composition Among Swimmers: A Systematic Review and Meta-Analysis of Randomized Controlled Trials
Source: Sports Med Open. 2024 Oct 23;10:115. doi: 10.1186/s40798-024-00784-8 (PMC11499511; doi:10.1186/s40798-024-00784-8)
Supplement: Supplementary file 2 — Supplementary Material 2 (Please replace 'Supplementary Material 2’ by the newly uploaded file 'Supplementary Material_2.pdf’ as there are changes in the reference numbers) [file 40798_2024_784_MOESM2_ESM.pdf]

Supplementary material_2

Article title:

Effects of Creatine Supplementation on the Performance, Physiological Performance, and Body Composition among Swimmers: A Systematic Review and Meta-Analysis of Randomized Controlled Trials

Journal name:

Sports Medicine Open

Author names:

Dongxiang HUANG[1,2], Xiaobing WANG[1], Tomohiro GONJO[3], Hideki TAKAGI[4], Bo HUANG[5], Wenrui HUANG[6], Qi SHAN[2], Daniel Hung-Kay CHOW[2]

Affiliation:

[1] School of Physical Education, Shaoguan University, Shaoguan, P.R. China.

[2] Department of Health and Physical Education, The Education University of Hong Kong, Hong Kong, P.R. China.

[3] Institute for Life and Earth Sciences, School of Energy, Geoscience, Infrastructure and Society, Heriot-Watt University, Edinburgh, United Kingdom.

[4] Faculty of Health and Sport Sciences, University of Tsukuba, Tsukuba, Japan.

[5] School of Physical Education and Sports Science, South China Normal University, Guangzhou, P.R. China.

[6] Shenzhen Traditional Chinese Medicine Hospital, Shenzhen, P.R. China

Corresponding author:

Prof. Dr. Hung Kay Daniel CHOW; Department of Health and Physical Education, The Education University of Hong Kong, Hong Kong, P.R. China; E-mail: danielchow@eduhk.hk

Table S1 Search results

Databases	ID	Searches	Results
Embase	#1	creatine'/exp	126322
	#2	'creatine hydrate':ti,ab,kw OR methylglycocyamine:ti,ab,kw OR 'methylguanidoacetic acid':ti,ab,kw OR 'n amidinosarcosine':ti,ab,kw OR 'n guanyl n methylglycine':ti,ab,kw OR 'n methyl n guanylglycine':ti,ab,kw OR 'cr supplementation':ti,ab,kw OR 'creatine loading':ti,ab,kw OR 'creatine monohydrate':ti,ab,kw OR 'creatine supplementation':ti,ab,kw OR 'oral creatine':ti,ab,kw	1803
	#3	'swimming'/exp	28962
	#4	swim:ti,ab,kw OR swimmer:ti,ab,kw OR freestyle:ti,ab,kw OR 'front crawl':ti,ab,kw OR 'back crawl':ti,ab,kw OR backstroke:ti,ab,kw OR breaststroke:ti,ab,kw OR 'butterfly stroke':ti,ab,kw OR 'butterfly swimming':ti,ab,kw OR fc:ti,ab,kw	102511
	#5	(#1 OR #2) AND (#3 OR #4)	**613**
PubMed	#1	swimming[MeSH Terms]	27,453
	#2	Swim[Title/Abstract] OR swimmer[Title/Abstract] OR Freestyle[Title/Abstract] OR "Front Crawl"[Title/Abstract] OR "Back Crawl"[Title/Abstract] OR Backstroke[Title/Abstract] OR Breaststroke[Title/Abstract] OR "Butterfly Stroke"[Title/Abstract] OR "Butterfly Swimming"[Title/Abstract] OR FC[Title/Abstract]	74,235
	#3	Creatine[MeSH Terms]	16,586
	#4	"Creatine hydrate"[Title/Abstract] OR "creatine monohydrate"[Title/Abstract] OR methylglycocyamine[Title/Abstract] OR "methylguanidoacetic acid"[Title/Abstract] OR "n amidinosarcosine"[Title/Abstract] OR "n guanyl n methylglycine"[Title/Abstract] OR "n methyl n guanylglycine"[Title/Abstract] OR "Cr supplementation"[Title/Abstract] OR "creatine loading"[Title/Abstract] OR "creatine supplementation"[Title/Abstract] OR "oral creatine"[Title/Abstract]	1,519
	#5	(#1 OR #2) AND (#3 OR #4)	**91**
Web of science	#1	TS=(Creatine OR “Creatine hydrate" OR "creatine monohydrate" OR methylglycocyamine OR "methylguanidoacetic acid" OR "n amidinosarcosine" OR "n guanyl n methylglycine" OR "n methyl n guanylglycine" OR "Cr supplementation" OR "creatine loading" OR "creatine supplementation" OR "oral creatine") and Preprint Citation Index (Exclude – Database)	110863
	#2	TS=(swimming OR Swim OR swimmer OR Freestyle OR "Front Crawl" OR "Back Crawl" OR Backstroke OR Breaststroke OR "Butterfly Stroke" OR "Butterfly Swimming" OR FC) and Preprint Citation Index (Exclude – Database)	382880
	#3	#2 AND #1 and Preprint Citation Index (Exclude – Database)	**999**

Table S1 (Continued)

Database	ID	Searches	Results
Scopus	#1	TITLE-ABS-KEY (Creatine OR “Creatine hydrate" OR "creatine monohydrate" OR methylglycocyamine OR "methylguanidoacetic acid" OR "n amidinosarcosine" OR "n guanyl n methylglycine" OR "n methyl n guanylglycine" OR "Cr supplementation" OR "creatine loading" OR "creatine supplementation" OR "oral creatine") AND TITLE-ABS-KEY (Swimming OR Swim OR swimmer OR Freestyle OR "Front Crawl" OR "Back Crawl" OR Backstroke OR Breaststroke OR "Butterfly Stroke" OR "Butterfly Swimming" OR FC)	**956**
Cochran Library	#1	('Creatine hydrate' OR 'creatine monohydrate' OR methylglycocyamine OR 'methylguanidoacetic acid' OR 'n amidinosarcosine' OR 'n guanyl n methylglycine' OR 'n methyl n guanylglycine' OR 'Cr supplementation' OR 'creatine loading' OR 'creatine monohydrate' OR 'creatine supplementation' OR 'oral creatine'):ti,ab,kw	2786
	#2	MeSH descriptor: [Creatine] explode all trees	940
	#3	(swim OR swimmer OR Freestyle OR 'Front Crawl' OR 'Back Crawl' OR Backstroke OR Breaststroke OR 'Butterfly Stroke' OR 'Butterfly Swimming' OR FC):ti,ab,kw	5461
	#4	MeSH descriptor: [Swimming] explode all trees	650
	#5	(#1 OR #2) AND (#3 OR #4)	**33**
SPORTDiscus	S1	SU (Swimming OR Swim OR swimmer OR Freestyle OR "Front Crawl" OR "Back Crawl" OR Backstroke OR Breaststroke OR "Butterfly Stroke" OR "Butterfly Swimming" OR FC)	13523
	S2	SU (Creatine OR “Creatine hydrate" OR "creatine monohydrate" OR methylglycocyamine OR "methylguanidoacetic acid" OR "n amidinosarcosine" OR "n guanyl n methylglycine" OR "n methyl n guanylglycine" OR "Cr supplementation" OR "creatine loading" OR "creatine supplementation" OR "oral creatine")	955
	S3	S1 AND S2	**13**

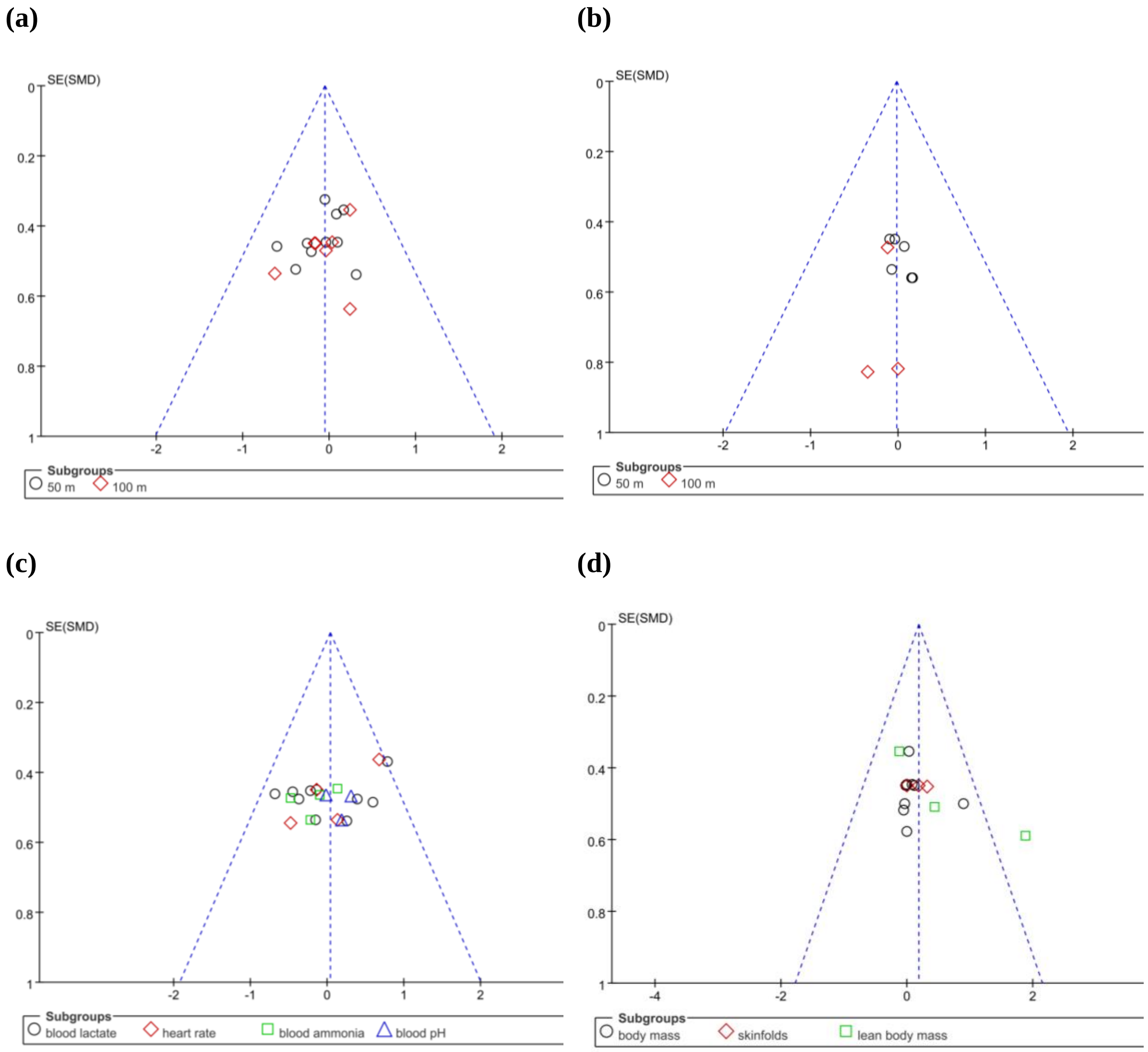

Fig. S1 Funnel plot for (a) single sprint swimming time, (b) repeated interval swimming time, (c) physiological variables, and (d) body composition

Study or Subgroup	creatine Mean	creatine SD	creatine Total	placebo Mean	placebo SD	placebo Total	Weight	Std. Mean Difference IV, Random, 95% CI
**1.4.1 body mass**								
Dawson et al. 2002 [10]	0.2	8.52	10	0.2	6	10	7.2%	0.00 [-0.88, 0.88]
Grindstaff et al. 1997 [18]	0.5	0.2	9	0.2	0.4	9	5.7%	0.90 [-0.08, 1.89]
Leenders et al. 1999 [19]	0.5	28.48	16	-0.6	24.31	16	11.6%	0.04 [-0.65, 0.73]
Mujika et al. 1996 [12]	0.7	9.95	10	-0.3	10.86	10	7.2%	0.09 [-0.79, 0.97]
Peyrebrune et al. 2005 (acute loading) [20]	-0.2	15.5	9	0	11.05	11	7.1%	-0.01 [-0.90, 0.87]
Peyrebrune et al. 2005 (maintenance) [20]	0.8	15.55	9	-0.8	10.69	11	7.1%	0.12 [-0.76, 1.00]
Selsby et al. 2003 [13]	0	9.65	8	0.5	5.43	7	5.4%	-0.06 [-1.07, 0.96]
Silva et al. 2007 [21]	-0.21	5.75	8	0.04	6.05	8	5.8%	-0.04 [-1.02, 0.94]
Theodorou et al. 1999 [24]	0.1	11.85	6	0.1	15	6	4.3%	0.00 [-1.13, 1.13]
**Subtotal (95% CI)**			**85**			**88**	**61.5%**	**0.11 [-0.19, 0.41]**
Heterogeneity: Tau² = 0.00; Chi² = 2.92, df = 8 (P = 0.94); I² = 0%								
Test for overall effect: Z = 0.69 (P = 0.49)								
**1.4.2 skinfolds**								
Dawson et al. 2002 [10]	-5.6	20.49	10	-9.8	22.67	10	7.2%	0.19 [-0.69, 1.07]
Peyrebrune et al. 2005 (acute loading) [20]	0.4	12.95	9	0.5	17.3	11	7.1%	-0.01 [-0.89, 0.87]
Peyrebrune et al. 2005 (maintenance) [20]	1.2	12.2	9	-3.5	14.83	11	7.0%	0.33 [-0.56, 1.22]
**Subtotal (95% CI)**			**28**			**32**	**21.4%**	**0.17 [-0.34, 0.68]**
Heterogeneity: Tau² = 0.00; Chi² = 0.28, df = 2 (P = 0.87); I² = 0%								
Test for overall effect: Z = 0.65 (P = 0.52)								
**1.4.3 lean body mass**								
Leenders et al. 1999 [18]	-2.1	13.19	16	-0.3	16.7	16	11.5%	-0.12 [-0.81, 0.58]
Silva et al. 2007 [21]	1.08	2.9	8	-0.3	3.07	8	5.6%	0.44 [-0.56, 1.43]
**Subtotal (95% CI)**			**24**			**24**	**17.1%**	**0.06 [-0.50, 0.63]**
Heterogeneity: Tau² = 0.00; Chi² = 0.80, df = 1 (P = 0.37); I² = 0%								
Test for overall effect: Z = 0.22 (P = 0.82)								
**Total (95% CI)**			**137**			**144**	**100.0%**	**0.11 [-0.12, 0.35]**
Heterogeneity: Tau² = 0.00; Chi² = 4.07, df = 13 (P = 0.99); I² = 0%								
Test for overall effect: Z = 0.93 (P = 0.35)								
Test for subgroup differences: Chi² = 0.08, df = 2 (P = 0.96), I² = 0%								

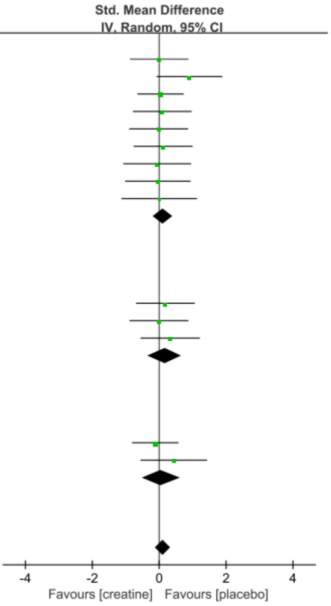

Fig. S2 Pooled analysis of the effect of Cr on body composition after a sensitivity analysis. Effects for the subgroups are based on the grouping variables of different outcomes (body mass vs skinfolds vs lean body mass)
